# Supplementary material for: Learning curve and surgical outcome of robotic assisted colorectal surgery with ERAS program
Source: Sci Rep. 2022 Nov 29;12:20566. doi: 10.1038/s41598-022-24665-w (PMC9709162; doi:10.1038/s41598-022-24665-w)
Supplement: Supplementary file 2 — Supplementary Information 2. [file 41598_2022_24665_MOESM2_ESM.zip › RAL Raw data-Ñ[▒K/110/HS_ñw╝╨░O▒Kñσ.pdf]

正式摘要(電子簽章：0000002255111047)		
W52 / W52 ☐ 英文標題(注意:無英文姓名-請補病人基本檔!)	☐【讀取】憑證	

臺中榮民總醫院

出院病歷摘要

轉入號 : 轉出號 :

[redacted]

**(8)**流水編號	
**(9)**入院日期	110/09/08 (2021) 直腸外科 W52 - 092
**(10)**轉科日期	
**(11)**出院日期	110/09/13 (2021) 住院天數計 5 日

診斷

- 入院 : 1. Adenocarcinoma, moderately differentiated, of Rectum, 2 cm FAV, cT3N1M0, - s/p neoadjuvant radiotherapy (IGRT, total 5000cGy in 25 fr.) 2021/05/20~06/25, under Ufur 2# BID
  2. Type II DM under regular medicine,(07/12 HbA1C:8.1)
- 出院 : 1. Adenocarcinoma, moderately differentiated, of Rectum, 2 cm FAV, cT3N1M0,
  - s/p neoadjuvant radiotherapy (IGRT, total 5000cGy in 25 fr.) 2021/05/20~06/25, under Ufur 2# BID
  - s/p robotic-assisted low anterior resection on 2021/09/09
  2. Type II DM under regular medicine,(07/12 HbA1C:8.1)

主訴

- blood-tinged stool for half year

病史

- This 55-year-old man has history of
  1. Adenocarcinoma, moderately differentiated, of Rectum, 2 cm FAV, cT3N0M0, s/p neoadjuvant RT 2021/05/20~06/25
  2. Type II DM under regular medicine,(07/12 HbA1C:8.1)

  He was suffered from hematochezia for half year, he had visited Tung's hospital and colonoscopy revealed rectal tumor at 2cm FAV, biopsy showed adenocarcinoma, moderately differentiated. He came to our hospital for second opinion on 20201/05/11. He underwent neoadjuvant radiation therapy during 2021/05/20~06/25. This time, he was admitted for scheduled robotic assisted low anterior resection.

體檢發現

-
  - General appearance :
    - easy looking , conscious clear , without cardiopulmonary distress
    - Vital signs自動帶入date 體溫(BT) 脈搏(P) 呼吸(R) 血壓(BP) 身高(BH) 體重(BW) BMI
      20210908 36℃ 79次/分 19次/分 152/119mmHg 168.9cm 61.3kg 21.5
    - Vital signs: B.P. 152/119 mmHg , P.R. 79 /min , R.R. 19 /min , B.T. 36 ℃
  - HEENT :
    - Eyes: no anemic conjunctiva, no icteric sclera, normal EOM
    - ENT: no otorrhea, no rhinorrhea, no erythematous mucosa, no injected tonsil, no deformity
  - Neck: supple, freely movable, trachea midline,no deformity, no engorged jugular vein , no palpable thyroid or lymph node enlargement
  - Chest:
    - Inspection: Grossly normal, symmetric and fully expansion, no accessory muscle used, no operation scar, no spider angiomata, no gynecomastia
    - Palpation: no palpable mass or nodule, no subcutaneous emphysema, no tenderness
    - Ausculation: clear breathing sound
  - Heart:
    - Percussion: normal heart size
    - Ausculation: regular heart beat, normal S1, S2, no gallop, no murmur

-
  - Abdomen:
    - Inspection: in
      - flat contour, symetrically, no scar, no stria, no dilated veins, no rashes
    - Ausculation: normoactive bowel sound, no abdominal bruits
    - palpation: soft, no muscle guarding or rigidity, no local tenderness, no rebounding pain, no palpable mass, no palpable pulsation
    - Percussion: no shifting dulllness, no hepatomegaly, no splenomagaly
  -

Peripheral pulsation :	CA	BA	RA	FA	PA	PTA	DPA
Right side :	++	++	++	++	++	++	++/+++
Left side :	++	++	++	++	++	++	++/+++

  - Genito-anus: no tenderness or palpable mass lesion or rectal shelf, normal external genitalia, normal anal tone, brown stool

CRS CONDITION

- Colonoscope report: UP TO 3CM FAV.RECTAL TUMOR GETS SMALLER. RECTAL CNCER S/P CCRT
- CEA: 3.54 ng/ml
-
  - Lung

手術日期及方法

- 手術日期及方法20210909 1. Robotic (XI) assisted low anterior resection
  2. Intraoperative colonoscopy : (-).
  3. High ligation (+) : At IMA root (Via robotic clips). IMV was ligated via endoclip.
  4. Take down from the splenic flexure : (-)
  5. Robotic EndoGIA (45-4.3) X2 + EEA (33mm) + OpenGIA (80-3.8) X1 were applied to complete the side-to-end anastomosis
  6. Proximal cut end: > 10 cm, distal cut end: 1.5 cm
  7. No drainage tube was inserted
  8. ADHESION BARRIER FILM was placed
  9. Loop ileostomy (About 15~20cm from IC valve) was done over RLQ of abdomen
  # Notice : While firing the second staple of the robotic endoGIA, malfunction was detected. Special device was applied to release the GIA. A new instrument was used instead, under the safety concern. The third robotic endoGIA was then fired successfully.
- 手術結果及發現 :
  - 1. A 2 x2 cm tumor at lower rectum. (DRE : Finger tip could reach the lower edge of the tumor) 2. No other gross tumor nor polyps was found 3. There was no obvious visible liver metastasis, no visible peritoneal seeding 4. No obvious ascites was noted 5. Bilateral doughnuts : intact ; Air leak test: Done and no air leak was found

住院治療經過

- After admission, preoperative prepare according to ERAS protocol was done, he underwent robotic-assisted low anterior resection smoothly on 2021/09/09. After surgery, he had regained conscious and tolerated well under postoperative ERAS protocol. Under his relative stable condition, he was discharged.

合併症

- Nil

一般檢查

- 累積報告 :

DATE	TIME	ALB	TP	BIL.T	BIL.D	ALKP	AST	ALT	LDH	AMY	LIPASE	NH3	Ferritin	UA
1100908	1943	5.1		2.03	0.44		43	44						
1100909	1457	3.4		1.28	0.39		34	34						

DATE	TIME	WBC	RBC	HGB	HCT	MCV	PLT	BLST	NEUT	BAND	SEG	LYM	MON	EOS	BAS
1100908	1802	3360	3.32	12.8	36.1	108.7	192		60.1			27.7	7.7	3.9	0.6
1100909	1457	7590	2.98	11.7	31.7	106.4	155		88.2			6.2	5.3	0.3	0.0

DATE	TIME	NA	K	CL	BUN	CREAT	eGFR	CA	Mg	P	CRP	ESR	PCT	D-Dimer	LACTATE
1100908	1943	142	3.8		21	2.21	33.02	9.2							
1100909	1457	138	3.6		13	1.20	66.81	7.6							

DATE	TIME	GLUCOSE	HbA1C	CHOL	TG	HDL	LDL	KETONE	CK	CKMB	TRO-T	PT-P	APTT-P	NT-pro BNP
1100908	1802	132												
1100908	1803		6.9											

DATE	TIME	PH	BEB	SCVO2	PO2	HCO3	HGB	PCO2	TCO2
1100909	1455	7.348	-3.3	97.8	108.9	22.1		41.2	23.4

-

特殊檢查

病理報告

- 活體組織切片病理報告　手術日期：**110/09/09**　病理號：**S1028426**

診斷醫師:李欣倪 報告日期:110/09/15
Pathologic diagnosis:
1. Rectum, robotic low anterior resection --- Status post-neoadjuvant radiotherapy with no residual invasive tumor. Ulceration and focal mild mucosal dysplasia are noted. The bilateral cut ends and perirectal lymph nodes (0/12) are free of tumor.
2. Rectum, cut end, distal, resection --- Chronic inflammation.
3. IMA root, dissection --- Unremarkable fibrovasculoadipose tissue.

Prognostic and predictive factor:
1. Tumor size: No residual tumor found.
2. Depth of invasion: No residual tumor found.
3. Histologic grade: Not applicable.
4. Angiolymphatic invasion: Not identified.
5. Perineural invasion: Not identified.
6. Tumor deposits (discontinuous extramural extension): Not identified.
7. Macroscopic tumor perforation: Not identified.
8. Surgical Margins: a) Proximal Margin: Uninvolved by invasive carcinoma. b) Distal Margin: Uninvolved by invasive carcinoma. c) Radial or Mesenteric Margin: Uninvolved by invasive carcinoma.
9. Lymph node status: Uninvolved (0/12).
10. Extranodal involvement: Not identified.
11. Tumor regression grading S/P CCRT: Present, no viable cancer cells (complete response, score 0) (neoadjuvant radiotherapy in this case).
12. Type of polyp in which invasive carcinoma arose: Not applicable.
13. Additional pathologic findings: Not identified.
14. Pathological TNM Stage: ypT0N0(According to the eighth edition, American Joint Committee on Cancer Staging Guidelines for Tumors).
15. TNM descriptors: y (Post-treatment).

Gross description:
The specimen consists of 1) a segment of colon, 13 cm in length and 4.5 cm in proximal cut end circumference, 6.5 cm in distal circumference, and perirectal soft tissue. A gray tan firm annular elevated and centrally ulcerative lesion, 1.5x1 cm, is noted, 0.8 cm to the distal cut end. On cross section, the lesion confined to the mucosa with 1.5 cm to the radial margin. The both cut ends are free of lesion grossly. 2) distal cut end, a piece of tan mucosa-covered soft tissue, 2x0.5x1 cm. 2) IMA root, a piece of tan yellow soft tissue, 1.5x0.5x0.2 cm. Representative part for section: A) proximal cut end B-F) tumor with distal cut end G) specimen 2 H) all of specimen 3 J-K) mesocolic lymph nodes.
#T-59600_2 #M-09413_2 0 1105 00SP00
#T-C4560_2 #M-09183_2 0 1105 000000

其它

出院時情況

- 3.改門診治療

出院指示

藥名	劑量	劑量單位	用法	頻次	數量	數量單位	現況	天數	單位
Norvasc tab 5mg	1	TAB	PO	QD	9	TAB	TH	9	天
(口)Takepron OD tab 30mg	1	TAB	PO	QDAC	9	TAB	TH	9	天
Ultracet tab	1	TAB	PO	Q6H	36	TAB	TH	9	天
Mag. Oxide tab	2	TAB	PO	TID	54	TAB	TH	9	天
Amamet F.C. tab 2/500mg	1	TAB	PO	QDAC	9	TAB	TH	9	天

出院計劃

- 門診日期
  - date on 2021/09/22
- 衛教 :一、飲食攝取：1.少量多餐。2.禁食刺激性食物，如太辣、太腥，大量飲酒及抽太多煙、濃茶和咖啡。3.多攝取高蛋白食物，以加速傷口修復。如：新鮮瘦肉、魚、蛋等。4.並減少吃產氣食物可避免脹氣。如：牛奶、豆類、地瓜、蛋、洋蔥。二、術後活動：1.三個月內不可從事下列活動：騎馬、騎腳踏車、劇烈的跳舞、久坐及開車，以免骨盆充血。2.手術後６？８週內避免提超過5KG之重物，若提物品可使用腹托或束腹帶以免震動所引起之疼痛。3.依個人的體力採漸進增加活動量，可先採輕工作，如無不適，體力許可再增加活動量。三、傷口護理：1.保持傷口清潔、乾燥，若有不慎弄濕傷口，可用清潔的毛巾或紗布拭乾。2.每日觀察傷口，是否有紅、腫、熱、痛，或不正常的分泌物。四、居家服藥：1.帶回的藥物，注意藥袋上的服用劑量、時間及注意事項。2.服用軟便劑若解便正常或有腹瀉現象時應停藥或減量。 五、出院後，若有下列情況需立即返院檢查：1.高燒不退。2.腹部疼痛厲害或嚴重腹脹嘔吐。3.傷口流血不止，或是有不正常之分泌物4.有其它問題，請與大腸直腸外科相關人員聯絡 TEL: 04-23592525 轉5161-3 或 6051, 6052

主治醫師 2062F 蔣鋒帆 / 住院醫師 2336L 洪浚嚴

正式摘要(電子簽章：000000225511047)

W52 / W52

doctorRank:B
